# Number processing and arithmetic skills in children with cochlear implants

**DOI:** 10.3389/fpsyg.2014.01479

**Published:** 2014-12-16

**Authors:** Silvia Pixner, Martin Leyrer, Korbinian Moeller

**Affiliations:** ^1^Institute of Applied Psychology, UMIT – The Health and Life Sciences UniversityHall in Tyrol, Austria; ^2^Department of Otolaryngology, Paracelsus University Medical School SalzburgSalzburg, Austria; ^3^Department of Linguistics, University of SalzburgSalzburg, Austria; ^4^Knowledge Media Research CenterTübingen, Germany; ^5^Department of Psychology, University of TübingenTübingen, Germany

**Keywords:** number processing, multiplication, number line estimation, subtraction, cochlear implants

## Abstract

Though previous findings report that hearing impaired children exhibit impaired language and arithmetic skills, our current understanding of how hearing and the associated language impairments may influence the development of arithmetic skills is still limited. In the current study numerical/arithmetic performance of 45 children with a cochlea implant were compared to that of controls matched for hearing age, intelligence and sex. Our main results were twofold disclosing that children with CI show general as well as specific numerical/arithmetic impairments. On the one hand, we found an increased percentage of children with CI with an indication of dyscalculia symptoms, a general slowing in multiplication and subtraction as well as less accurate number line estimations. On the other hand, however, children with CI exhibited very circumscribed difficulties associated with place-value processing. Performance declined specifically when subtraction required a borrow procedure and number line estimation required the integration of units, tens, and hundreds instead of only units and tens. Thus, it seems that despite initially atypical language development, children with CI are able to acquire arithmetic skills in a qualitatively similar fashion as their normal hearing peers. Nonetheless, when demands on place-value understanding, which has only recently been proposed to be language mediated, hearing impaired children experience specific difficulties.

## INTRODUCTION

At first glance, skills like mental arithmetic and/or magnitude comparison are not readily dependent on language abilities. In fact, quite many numerical competencies – such as numerical discrimination and additive/subtractive expectations – are mastered well before children are able to actively produce their first words (for a respective review, see Feigenson et al., 2004). Thus, the lack of studies investigating numerical cognition in individuals with impaired hearing (who are known to have a delayed or even aberrant language development) comes to no surprise. The present study aims to address the case of deaf and profoundly hearing impaired children who received a cochlear implant (CI) during early infancy.

Recent research shows unambiguously that early implantation enables most of the young users with CI to develop considerable speech and language competence (e.g., Geers et al., 2003; Nott et al., 2003; Tomblin et al., 2005; Connor et al., 2006; Johnson and Goswami, 2010). The developmental trajectories, however, seem to be deviant from typical with respect to phonetics and phonology and delayed with respect to grammar and lexicon (e.g., Blarney et al., 2001; Chin, 2006; Leyrer, 2008; Adi-Bensaid and Tubul-Lacy, 2009; Geers et al., 2009; Friedmann and Szterman, 2011). Importantly, such variability in developmental pathways of linguistic skills might affect other cognitive domains such as numerical cognition. In the present study, we will evaluate the benefits and limitations of cochlear implantation for the acquisition of numerical skills in affected children. Before presenting the experimental study the interrelation of numerical and language skills will be elaborated on briefly.

### THE INTERRELATION OF NUMERICAL AND LANGUAGE SKILLS

It has been argued that language plays a key role in the development of number-related language processing and in particular so in the development of number concepts (e.g., Carey, 2004). Following this, LeFevre et al. (2010) proposed a developmental calculation model that differentiates three relevant pathways for the development of numerical skills: a linguistic pathway, a quantitative and a spatial attention pathway. In their sample of 182 children aged 4.5–7.5 years the three aforementioned pathways were found to contribute independently to early numerical skills. Moreover, there is also evidence suggesting influences of language skills to be rather specific. The currently most influential model of number processing – the Triple Code model _(see Dehaene et al., 2003; Arsalidou and Taylor, 2011 for latest amendments) – suggests numerical information to be represented by three codes within the human brain. The visual-Arabic number form is the most basic code. It is recruited to perceive digits as numerically informative symbols and associated with bilateral occipital brain areas. Additionally, the Triple Code model differentiates between an analogue quantity code and verbal numerical representations. The analogue quantity code is involved whenever quantity or magnitude information of numbers is processed. It is assumed to be subserved by bilateral cortex areas around the intra-parietal sulcus. Finally, verbal numerical representations are recruited in tasks such as number naming. Additionally, arithmetic facts (e.g., multiplication fact knowledge) are assumed to be stored in a verbal code. Verbal numerical representations are associated with left-lateralized perisylvian language areas and the angular gyrus. In line with the assumption of a verbal numerical representation language influences should be most prominent when it comes to arithmetic fact knowledge, whereas representations of numerical quantity and numerical symbols should be less dependent on language. Noteworthy, the findings of Koponen et al. (2006) revealed that number naming speed is indeed closely related to arithmetic fact retrieval. This means that skilled calculators are able to directly retrieve the result of a number fact (e.g., 3 × 4) from phonological long-term memory without having to apply procedural calculation strategies. Additionally, it has been observed that this fact retrieval processes are subject to interference by concurrent articulation (Lee and Kang, 2002; Moeller et al., 2011). This provides further evidence for the verbal (language related) format of arithmetic fact knowledge. Nevertheless, also more direct evidence for the close interrelation between number fact retrieval and language abilities is accumulating. For instance, Fazio (1999) reported deficient fact retrieval skills in children diagnosed with a specific language impairment (SLI, see also Donlan et al., 2007). Furthermore, it has been found repeatedly that children suffering from dyslexia (whose core difficulty by definition is impaired acquisition of written language) often exhibit deficient number fact retrieval, too (e.g., Snowling, 2000; see also Miles et al., 2001; Simmons and Singleton, 2008; De Smedt and Boets, 2010; Göbel and Snowling, 2010). Finally, beyond the case of fact retrieval, counting abilities have also been observed to be associated with language competencies (children with dyslexia: Simmons and Singleton, 2008; children with SLI: Koponen et al., 2006; Donlan et al., 2007).

Nevertheless, as already indicated in the Triple Code model not all aspects of numerical cognition should be associated with language competencies. In line with this suggestion it was found that subitizing (LeFevre et al., 2010), symbolic calculation (McNeil and Burgess, 2002), number comparison (O’Hearn and Luna, 2009) as well as number line estimation (requiring children to estimate the position of a given number on a presented number line; Koponen et al., 2006) are rather independent from language skills. However, even when these tasks are referred to as being non-verbal, recent research indicated that this might only be part of the story. For instance Pixner et al. (2011a) disclosed language influences on a two-digit number comparison task that was administered to German, Italian and Czech-speaking first graders. The three groups are ideal populations to study language influences because they are distinguishable regarding the correspondence between symbolic Arabic and spoken number word systems. While spoken and the symbolic Arabic number systems closely correspond to each other in Italian (venti-cinque/twenty-five → 25], the German number word system is intransparent insofar as the order of tens and units is inversed in spoken as compared to symbolic notation (fünfundzwanzig/five-and-twenty → 25). Finally, in Czech both inverted and non-inverted number words are utilized. Noteworthy, the intransparency of the inverted number word system posed particular difficulty on German-speaking children’s transcoding performance in general (Zuber et al., 2009) and Czech-speaking children when asked to transcode inverted number words (see also Pixner et al., 2011a for language effects in magnitude comparison). In line with this, mental number line representations also seem to be moderated by language characteristics (Helmreich et al., 2011). LeFevre et al. (2010) propose in their model of numerical development that the number line task calls on both semantic (number magnitude) and spatial representations. Additionally, the authors suggest that performing the number line estimation task for multi-digit number ranges requires mastery of the base-10 structure of the Arabic number system (see also Moeller et al., 2009) which in turn is clearly language dependent as indicated by recent evidence from transcoding (Zuber et al., 2009; Pixner et al., 2011b) but also number line estimation (Helmreich et al., 2011). Taken together, though there is accumulating evidence for a link between language and number processing knowledge on the exact nature and the underlying mechanisms of this association is still rather patchy even in typically developing not to say in atypically developing children such as deaf or children with CI.

### NUMBER PROCESSING IN HEARING IMPAIRED AND DEAF INDIVIDUALS

A frequent observation in educational settings is that children with profound hearing impairments as well as deaf children quite often experience difficulties to acquire calculation skills (e.g., Zarfaty et al., 2004; Ansell and Pagliaro, 2006). Upon taking into account the aforementioned link between language and numerical skills (typically developing children: Zuber et al., 2009; Helmreich et al., 2011; Pixner et al., 2011a,b; children with dyslexia: Simmons and Singleton, 2008; children with SLI: Koponen et al., 2006; Donlan et al., 2007) number-related deficiencies in hearing impaired individuals come to no surprise. Interestingly, already the National Council of Teachers of the Deaf Research Committee, 1957 examined 200 deaf students in Great Britain and found a significant (i.e., a 1–21/2 year) delay in the acquisition of arithmetical skills. Similar findings are reported by Kramer (2007) who investigated German-speaking deaf individuals. However, different from research on typically developing children for which the interrelation of language and number processing has been investigated quite specifically, there is a scarcity of studies systematically investigating specific numerical skills (e.g., arithmetic fact retrieval, basic arithmetic, number line estimation, etc.) and its relation to language skills for hearing impaired individuals in a comparative manner. Therefore, the present study pursued this issue.

### THE PRESENT STUDY

The main aim of the present study was to systematically examine numerical and arithmetical skills in formerly deaf children that received a CI in early childhood as compared to typically developing children. It is important to note that there is broad consensus in the literature indicating atypical language development in children with CI (e.g., Boothroyd et al., 1991; Dawson et al., 1995; Geers et al., 2009; Pisoni et al., 2010; Ingvalson and Wong, 2013; Rinaldi et al., 2013). Therefore, we did not wish to evaluate the influence of an atypical language development in children with CI on their numerical/arithmetic abilities by correlating their performance in a language task with those in numerical/arithmetic tasks. Instead, we compared the performance of children with CI and a control group matched on hearing age and general intellectual functioning in specific numerical/arithmetic tasks chosen for either their strong dependence on language-related processing (i.e., multiplication fact retrieval) or their only weak dependence on language skills (i.e., two-digit subtraction and number line estimation). Thereby, we aimed at evaluating whether impairments possibly observed for CI children may be associated with their known atypical language development. In particular, the following research questions and hypotheses were pursued.

(1) On a general level we were interested in whether the number of students with poor arithmetic skills is comparable for children with CI and their normal hearing (NH) peers. Following the rationale that numerical competencies are directly (arithmetic facts) or indirectly (magnitude representation) related to verbal language representations it is expected that children with CI are more likely to score poor on a standardized mathematics performance test as compared to their hearing peers.
(2) More specifically, it was of interest whether possible impairments of children with CI would be most pronounced for those tasks for which the closest association with language is assumed. When this is the case, children with CI should perform significantly worse than their hearing peers in a multiplication task assessing verbally driven arithmetic fact retrieval. On the other hand, for tasks with a more indirect association with language processes such as subtraction and/or number line estimation our hypotheses need to be more specific. Generally, children with CI should not perform worse on subtraction. However, this might be moderated by the need for a borrowing. Following the model of LeFevre et al. (2010) mastery of the place-value structure of the Arabic number system is language dependent (see also Pixner et al., 2011a,b). Thus, as successful application of the borrowing procedure requires place-value understanding (see Klein et al., 2010a,b for the case of carry in addition) we expect children with CI to experience particular difficulties for borrowing subtraction problems. Similarly, LeFevre et al. (2010) suggest performance in the number line estimation task to be associated with place-value understanding (see also Moeller et al., 2009; Helmreich et al., 2011). Thus, children with CI should also show poorer performance in the number line estimation task as compared to their NH peers.

## MATERIALS AND METHODS

### PARTICIPANTS

Overall, 94 children participated in the present study, 45 children (26 males) with CI and 49 NH controls (23 males). Cochlear implants were surgically placed when children were at a minimum of eight and a maximum of 50 months of age. Participating children attended third to fifth grade. Fourteen of the hearing impaired participants were in special educational schools. In these schools children with CI are taught together with deaf children. Even though it is tried to teach children with CI using spoken language as far as possible part of the instruction also includes sign language.

Though children with CI were older than their hearing peers, the two groups were comparable with respect to hearing experience and grade level (see **Table 1**). Please note that we used hearing age instead of age at implantation as a variable describing the hearing/language experience of the children with CI because it is possible to match the two groups on this variable. As we aimed at matching our two groups as closely as possible we decided to use hearing age because it is not possible to match the two groups on age at implantation as a measure of hearing/language experience.

**Table 1:** Demographic variables and background information on the study groups (mean ± SD).

	Children with CI *n* = 45	Control group *n* = 49	Statistical difference
Age (months)	122.18 ± 11.58	101.86 ± 16.29	*t*(92) = 6.94; *p* < 0.001
Hearing experience (months)	98.78 ± 15.35	101.86 ± 16.29	*t*(92) = 1.55; *n.s.*
Grade placement	Third grade *n* = 17 Fourth grade *n* = 21 Fifth grade *n* = 7	Third grade *n* = 20 Fourth grade *n* = 29	*t*(92) = 1.49; *n.s.*
Intellectual functioning (T-score)∘	53.00 ± 9.28∘	52.69 ± 4.81^§^	*t*(92) < 1; *n.s.*
Verbal working memory (forward span)^#^	4.93 ± 0.91	4.71 ± 0.68	*t*(92) = 1.33; *n.s.*
Central executive (CE) functioning (backward span)^#^	3.80 ± 0.89	2.92 ± 0.67	*t*(92) = 5.43; *p* < 0.001

*∘Measured by CFT 20-R ( Weiß, 2008)*.

*^§^ Measured when children attended first grade by the CFT-1 (Cattell et al., 1997). As the CFT is thought to tap the g-factor of intelligence (that is considered to be rather stable across development) we are confident that intellectual functions are comparable between participant groups*.

*^#^Measured by a letter span task*.

Moreover, in Austria hearing impaired children are likely to start school with a delay of one to 2 years. Thus, grade level was used as matching criteria between the experimental (CI) and the control group (NH) because grade level is more relevant (in terms of the received mathematical instruction) than chronological age when investigating arithmetical skills. Furthermore, the two study groups were comparable with respect to overall intellectual functioning and verbal working memory. For central executive (CE) functioning there even was an advantage for the Children with CI (see **Table 1**).

### ASSESSMENT AND PROCEDURE

The study was approved by the local ethics committee of the UMIT, Hall in Tyrol.

To assess *math achievement* children with CI had to complete the basic arithmetic operations scale of the Heidelberger Rechentest 1–4 (HRT 1–4; Haffner et al., 2005). Please note that control children were not administered the HRT.

Instead, both groups were asked to solve two PC-administered tasks tapping multiplication and subtraction skills. In addition, a number line estimation task was presented in paper-pencil format. Each child was tested individually in a separate room.

*Multiplication* capabilities were assessed by a verification task comprising 80 multiplication problems with one-digit operands. These critical trials were presented in randomized order preceded by 10 practice trials to ensure task comprehension. Stimuli were presented centrally on the screen in the form x × y = z (Arial font, size 48). On each trial, the problem was presented simultaneously with an either correct or incorrect solution probe. Additionally, incorrect probes were separated into two error types: operand errors representing the correct solution to a neighboring multiplication problem of the same table (e.g., 3 × 4 = 15) and so-called non-table errors not related to any item of the multiplication tables (e.g., 3 × 4 = 13). Children were asked to indicate by button press whether the solution probe was correct (right-hand button press) or not (left-hand button press). Stimulus presentation was preceded by a fixation cross presented at the center of the screen for 500 ms. Then the multiplication problem appeared and stayed visible until one of the response buttons was pressed. After an inter-stimulus-interval of 500 ms the fixation cross for the next trial was presented.

*Subtraction* skills were assessed by a choice reaction task involving 40 subtraction problems. Comparable to the multiplication task critical trials were presented in randomized order and were preceded by 10 practice trials. Importantly, in order to assess procedural solution strategies rather than direct fact retrieval (as dominant in multiplication) the subtraction task comprised two-digit numbers only. Problems were presented in the form xx – xx (Arial font, size: 48) at the x/y-coordinates (512/300) with the two solution probes appearing below the problem, either on the left side (x/y coordinates 300/550) or on the right side (x/y coordinates 724/550). Children had to single out the correct solution by button press (right or left button press). After a fixation cross was presented for 500 ms the problem and the solution probes appeared on the screen simultaneously. The stimuli stayed visible until one of the response buttons was pressed, directly followed by the fixation cross of the next trial. Subtractions were categorized into those requiring a borrow procedure (e.g., 52–37) and those not requiring a borrow procedure (e.g., 49–34). Importantly, problem size was matched between these item categories.

*Number line estimation* performance was assessed in a paper-pencil version of the number-to-position number line estimation task. Children were asked to estimate the spatial position of a given number on a number line ranging from 0 to 100 for a first set of items and from 0 to 1000 for a second set of items (each line measuring 10 cm). Only the start (0) and end (100 or 1000) point of the number line was specified by the respective Arabic number. Above each number line the target number was written in Arabic notation. Overall, 36 critical trials were presented (*n* = 18 per range) that were preceded by two practice items.

### SCORING AND ANALYSES

#### HRT

Performance in the standardized calculation test was scored according to the procedure described in the test manual (converting raw scores into T-scores).

#### Multiplication

Multiplication performance was analyzed in two separate two-way ANOVAs. In a first ANOVA the within-subject factor problem type (correct vs. incorrect) and the between subject factor participant group (CI vs. NH) were discerned. In the second ANOVA influences of the within-subject factor error type (operand vs. non-table error) and the between-subject factor participant group were evaluated. As the two participant groups differed reliably with respect to CE functioning this variable was incorporated as a covariate. Both analyses were conducted separately for reaction times (RT) and error rates.

#### Subtraction

Subtraction performance was evaluated using a two-way ANOVA incorporating the within-subject factor task borrowing (with vs. without) and the between-subject factor participant group (CI vs. NH). As for multiplication performance, this analysis was run for RT and error rates and CE functioning was considered as a covariate.

#### Number line estimation

Data analysis of the number line task considered the absolute estimation error (i.e., how far the actually indicated position deviated from the correct position of a target number). As two different number lines (i.e., ranging from 0 to 100 and from 0 to 1000) were employed in the current study and in order to make results comparable, the percent absolute estimation error [PAE; i.e., (target number – estimated number)/number range)] per number line range was used for further analyses (cf. Siegler and Booth, 2004). Finally, number line estimation performance in terms of PAE was evaluated in a two-way ANOVA with the within-subject factor number line range (0–100 vs. 0–1000) and the between-subject factor participant group (CI vs. NH). Again, CE functioning was incorporated as a covariate.

## RESULTS

### HRT

Seven out of 45 children with CI exhibited considerably poor performance on the index scale *basic arithmetic operations* (as indicated by a percentile <10). Please note that a percentile <10 is used as cut-off for the diagnostic criteria of developmental dyscalculia (International Classification of Diseases/ICD 10: Dilling and Freyberger, 2001; Diagnostic and Statistical Manual of Mental Disorders/DSM IV: American Psychiatric Association [APA], 1994). Hence, seven children of our experimental group (i.e., 15.6%) would fall below the diagnostic threshold of developmental dyscalculia. Interestingly, another seven children of the experimental group were found to exhibit excellent performance levels on the standardized calculation test (as indicated by percentiles >85). Nevertheless, an statistical evaluation indicated that the T-scores on the HRT index scale basic arithmetic operations of the experimental group (*M* = 48.40 SD = 10.49) did not differ reliably from those of the standardization sample of the HRT [*M* = 50.00, SD = 10.00; *t*(44) = 0.89, *n.s*.].

### MULTIPLICATION

#### Analyses of problem type

Analyses of error rates did not reveal any significant result. Neither the main effects of problem type or participant group nor their interaction turned out to be statistically reliable (all *F* < 1). There was also no significant influence of the covariate (*F* < 1).

**FIGURE 1:**
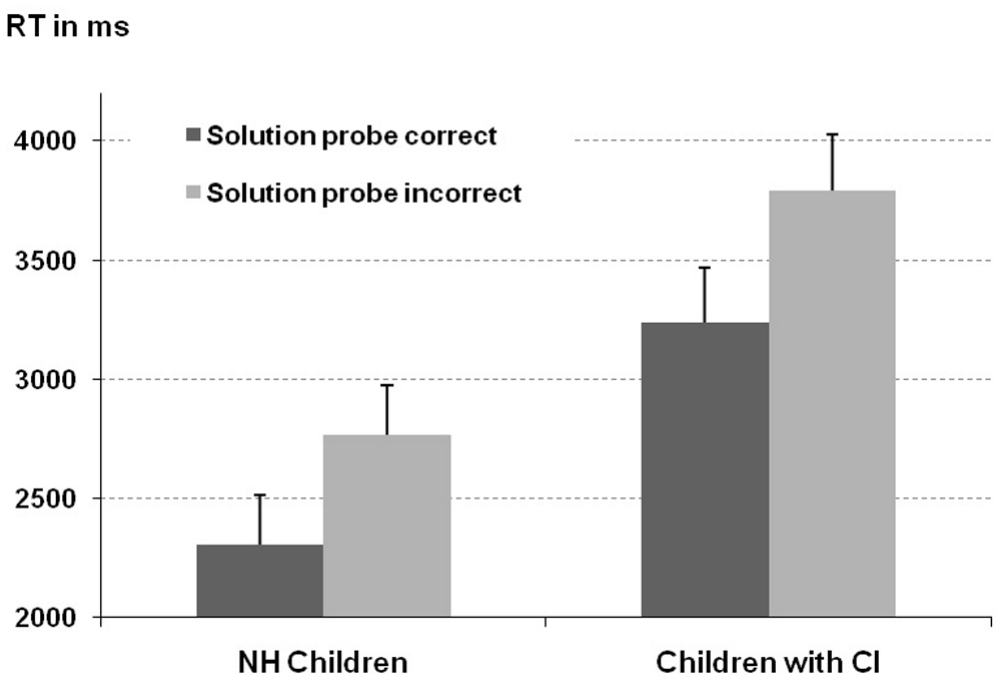
**Response latencies separated for problem type (presented with either correct or incorrect solution probe) and participant group.** Error bars represent one standard error of the mean (SEM).

With respect to response latencies the ANOVA revealed a significant main effect of problem type [*F*(1,91) = 14.61, *p* < 0.001]. This indicated faster responses for accepting a correct solution probe than rejecting an incorrect one (2773 ms vs. 3280 ms, respectively). Additionally, the reliable main effect of participant group [*F*(1,91) = 9.45, *p* < 0.01] indicated that latencies of children with CI were longer than those of NH controls (3516 ms vs. 2537 ms, respectively, see **Figure 1**). The interaction of problem type and group was not significant (*F* < 1). Finally, the influence of the covariate was significant [*F*(1,91) = 8.41, *p* < 0.01] with shorter latencies being associated with higher CE scores.

#### Analyses of error type

Again, the ANOVA on error rates did not reveal any significant result. Neither the main effects of error type or participant group nor their interaction turned out to be statistically reliable (all *F* < 1.25, all *p* > 0.27). Also the influence of the covariate was not reliable (*F* < 1).

The analysis of participants’ response latencies revealed a significant main effect of participant group [*F*(1,91) = 8.41, *p* < 0.01] indicating longer latencies for children with CI than for hearing controls (3794 ms vs. 2767 ms, respectively). Neither the main effect of error type nor the interaction of group and error type was statistically reliable (both *F* < 1). However, the influence of the covariate was significant [*F*(1,91) = 8.70, *p* < 0.01] associating higher CE scores with faster responses.

Taken together this indicates that responses of children with CI were reliably delayed. However, both groups exhibited comparable performance profiles (regarding problem type and error type).

### Subtraction

**FIGURE 2:**
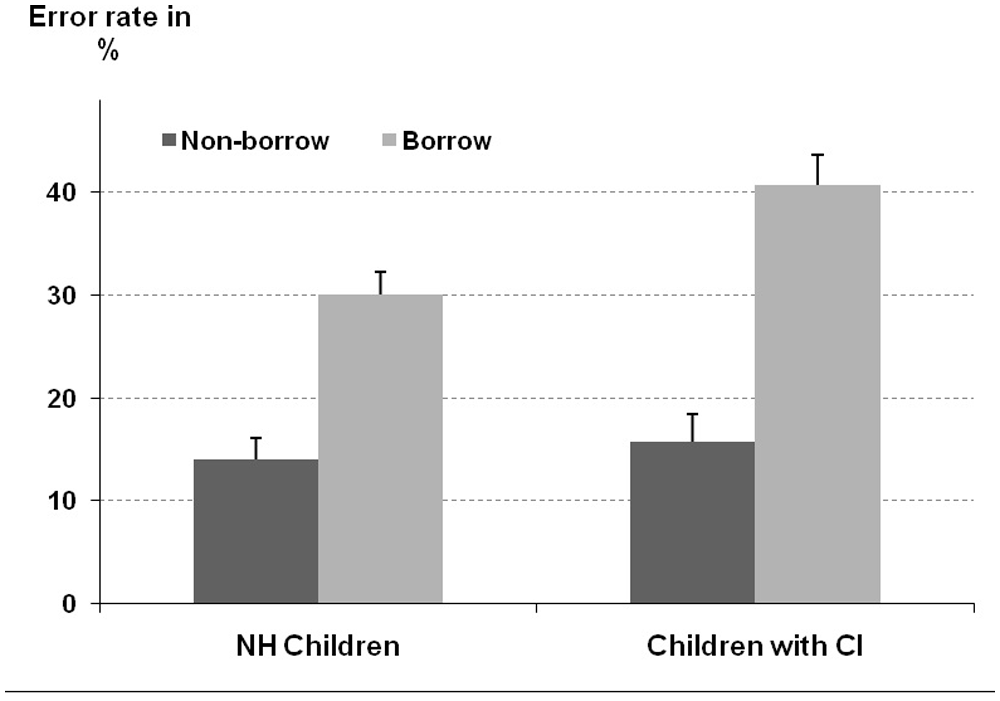
**Error rates for the subtraction task depicted separately for borrow conditions (non-borrow vs. borrow) and participant group.** Error bars reflect one SEM.

For error rates the ANOVA revealed a significant borrow effect [*F*(1,91) = 23.86, *p* < 0. 001]. As depicted in **Figure 2**, children committed significantly more errors on problems requiring a borrow than on problems not requiring a borrow procedure (35.4% vs. 14.8% errors, respectively). Moreover, the main effect of participant group was not reliable [*F*(1,91) = 2.82, *p* = 0.10] indicating that children with CI did not exhibit a significantly higher error rate than their NH peers (28.2% vs. 22.0% errors, respectively). Most importantly and in line with our expectations the significant interaction of borrow and participant group [*F*(1,91) = 4.82, *p* < 0.05] indicated that the borrowing effect was indeed more pronounced for children with CI as compared to NH controls (25.0% vs. 16.1% errors, respectively, see **Figure 2**). Finally, the influence of the covariate was reliable [*F*(1,91) = 6.03, *p* < 0.05] with a higher CE score being associated with a smaller error rate.

For response latencies only the main effect of participant group was reliable [*F*(1,91) = 12.82, *p* < 0.01]. This indicated that responses of children with CI responded were generally slower as compared to the responses of the control group (6691 ms vs. 5265 ms, respectively). Neither the main effect of borrowing nor the interaction of borrowing and participant group turned out to be reliable (both *F* < 1.26, both *p* > 0.26). Additionally, the influence of the covariate was not reliable [*F*(1,91) = 2.48, *p* = 0.12].

In summary, children with CI not only exhibited prolonged response latencies but also experienced difficulties when it comes to the specific processing of place-value information as required by subtraction problems incorporating a borrow procedure.

### Number line estimation

**FIGURE 3:**
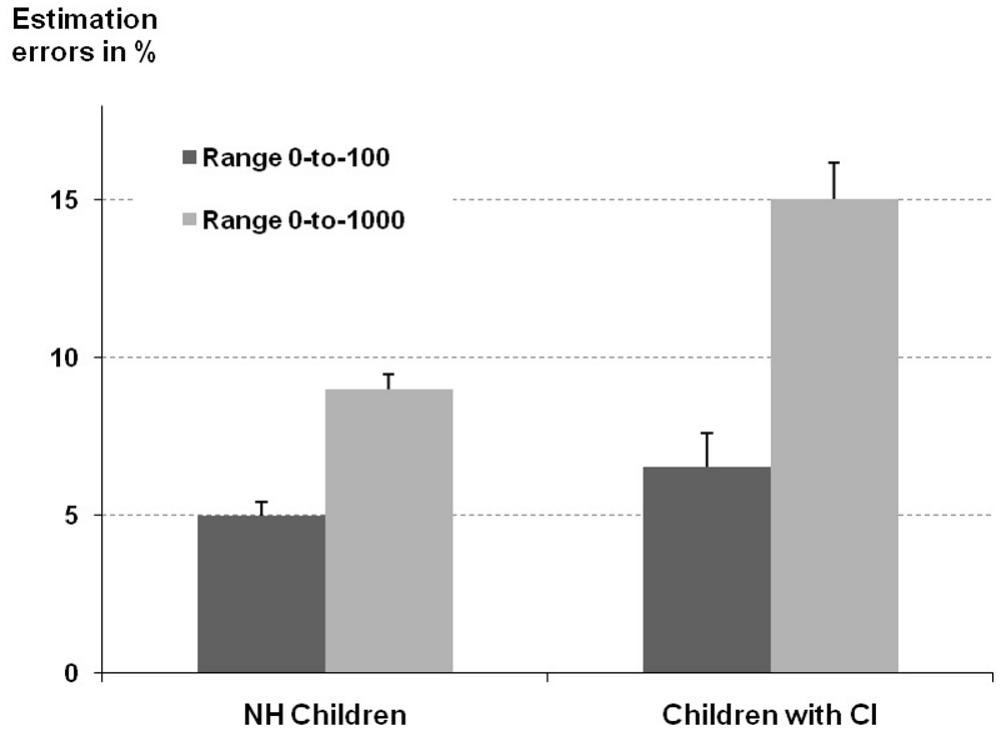
**Estimation error for the number line estimation task separated for number line range (0-to-100 vs. 0-to-1000) and participant group.** Error bars represent one SEM.

With respect to estimation errors the ANOVA revealed a significant main effect of number line range [*F*(1,91) = 32.30, *p* < 0.001]. This indicated that children’s estimation error was reliably larger when asked to mark the position of a given number on a number line ranging from 0 to 1000 as compared with those ranging from 0 to 100 (5.7% vs. 12.0% misplacement, respectively). Additionally, the main effect of participant group was also significant [*F*(1,91) = 11.76, *p* < 0.01]: compared to controls the estimation error of children with CI was significantly larger (10.8% vs. 7.0% misplacement, respectively). Moreover, these main effects were qualified by the reliable interaction of number line range and group [*F*(1,91) = 11.77, *p* < 0.01]. The interaction indicated that the increase of estimation error from the 0 to 100 to the 0 to 1000 number line range was more pronounced for children with CI than for hearing controls (8.5% vs. 4.2% increase in misplacement, respectively, see **Figure 3**). Finally, the influence of the covariate was significant [*F*(1,91) = 8.09, *p* < 0.01] indicating that higher CE scores were associated with a smaller estimation error, this means more precise localization of numbers on the number lines.

Summarizing the results for the number line estimation task it has to be noted that as for the subtraction task we observed specific impairments for children with CI as the demands on processing place-value information increased (i.e., from two- to three-digit numbers).

## DISCUSSION

The main aim of the present study was to investigate numerical/arithmetical capabilities in children with CI and to contrast their performance to NH peers of matched hearing age. We were interested in general as well as in specific performance differences between these participant groups. On the general level we expected children with CI to perform reliably worse than their NH peers, as arithmetical capabilities have been shown to be related to language representations and their processing both directly (e.g., arithmetic facts) and/or indirectly (e.g., magnitude representation). Additionally, we hypothesized that children with CI should be specifically impaired on arithmetical competencies with a specific reference to place-value understanding. According to LeFevre et al. (2010) the representation of the place-value structure of the Arabic number system is closely related to language representations (see also von Aster and Shalev, 2007 for a similar view) even in tasks usually considered not to address language-based numerical representations such as subtraction or number line estimation. Generally, the current data corroborated both of our hypotheses. We observed general (i.e., increased rates of dyscalculia indications, prolonged overall RT) as well as specific impairments (i.e., more pronounced borrowing effect) for children with CI. These will be discussed in turn in the following.

### GENERAL NUMERICAL IMPAIRMENTS OF CHILDREN WITH CI

In a first step, arithmetical skills of children with CI were examined by administering a standardized calculation test. Results revealed that 15.6% of children with CI exhibited performance levels being indicative of developmental dyscalculia. With a general prevalence rate of dyscalculia in the general school population of 4–7% (e.g., Badian, 1993; Gross-Tsur et al., 1996; von Aster et al., 2007) indications of dyscalculia were more prominent in children with CI than was to be expected. This increased rate of dyscalculia indications may be interpreted to index a general impairment of arithmetical capabilities in children with CI. Importantly, the overall result pattern for the more specific arithmetical assessment (i.e., multiplication, subtraction, and number line estimation) corroborated such a conclusion. For each of these tasks we observed reliable group differences indicating that children with CI performed more poorly than the hearing controls: children with CI took longer to complete the multiplication verification as well as the subtraction choice reaction task. Moreover, their number line estimations were less accurate as compared to those of the hearing controls. This indicated that children with CI seemed to suffer from a general impairment, in particular, a general slowing combined with reduced accuracy of mental number line representations, as regards their arithmetical capabilities. Taken together, this suggests that the known atypical language development of children with CI (e.g., Boothroyd et al., 1991; Dawson et al., 1995; Geers et al., 2009; Pisoni et al., 2010; Ingvalson and Wong, 2013; Rinaldi et al., 2013) seemed to exhibit a reliable negative influence on their numerical development as previously proposed (e.g., Fazio, 1999; Koponen et al., 2006). This is in line with recent evidence suggesting language to influence numerical tasks as basic as magnitude comparison and number line estimation but also more complex arithmetic in children (e.g., Helmreich et al., 2011; Pixner et al., 2011a,b; Göbel et al., 2014). Usually, it is argued that this is due to a coactivation of verbal numerical representations such as number names when children perform symbolic numerical tasks. This activation may occur automatically, but especially children are regularly found to verbalize numerical tasks to assist processing. When no or only impaired such coactivation of verbal numerical representations and thus verbalizing is possible because of impaired language abilities (as in children with CI) this may lead to the prolonged processing times.

However, closer inspection of the performance pattern of children with CI indicated that this might only be part of the story. On the one hand, 15.6% of children with CI were found to perform significantly above average on the HRT. Interestingly, this may indicate that the normal distribution describing arithmetic performance capabilities of children with CI may be flatter and broader as compared to that of normally developing children. However, the two distributions did not differ regarding their mean as indicated by our analyses. On the other hand, poorer performance in the HRT and the observed general slowing may be associated. The HRT as used in this study is a speeded test and the observed general slowing might have led to the increased number of dyscalculia indications in the sample of children with CI. Therefore, it is inevitably necessary not only to look at these general performance impairments but also to evaluate more specific performance differences associated with particular numerical competencies and/or representations.

### SPECIFIC IMPAIRMENTS OF CHILDREN WITH CI

Evaluating the specific impairments will consider the results of the computerized calculation tasks as well as the number line estimation task. While multiplication performance has repeatedly been suggested to be closely related to language skills (Lee and Kang, 2002; Dehaene et al., 2003; see also Fazio, 1999; Koponen et al., 2006), language is generally assumed to play either no or only a minor role for solving subtractions (of either one- or multi-digit operands; McNeil and Burgess, 2002) or number line estimation (e.g., Siegler et al., 2010; but see LeFevre et al., 2010). However, even though Lee and Kang (2002) and Moeller et al. (2011) report experimental evidence for impairments of multiplication fact retrieval due to a verbal secondary task, no other specific language related impairments for multiplication have been reported in the literature. Thus, despite a generally poorer multiplication performance we did not expect any further specification of performance differences. In line with this, we observed no differences between children with CI and NH controls with regard to the processing of correctly or incorrectly solved multiplication problems as well as for the differentiation between table and non-table errors. However, closer inspection of the association of hearing age with error types revealed an interesting result with respect to non-table errors. A correlation analysis indicated that the percentage of non-table errors was correlated significantly with hearing age in children with CI [*r*(45) = -0.30, *p* < 0.05] indicating that with increasing hearing age fewer non-table errors were committed. Interestingly, this correlation was not reliable in children without CI [*r*(49) = 0.11, n.s.]. Fisher’s *Z*-test indicated that the difference between the two correlations was significant (*Z* = 1.97, *p* < 0.05). Importantly, this pattern of results is in line with the assumption that impaired hearing/language experience of children with CI may have influenced their numerical development. Generally, multiplication is assumed to be solved via verbally mediated retrieval of arithmetic facts from long-term memory (e.g., Dehaene et al., 2003). Butterworth et al. (2003) found that with increasing skill level (i.e., automaticity of fact retrieval) the number of non-table errors decreased (see also Campbell and Graham, 1985). This is exactly what we observed for hearing/language experience of children with CI. Taken together, this indicated that the children with CI seem to process multiplication problems in a qualitatively similar way to hearing controls but with a quantitative difference arguing for an impairing influence of their reduced hearing/language experience. This interpretation was substantiated by the results for subtraction and number line estimation.

Based on the considerations of LeFevre et al. (2010; see also Helmreich et al., 2011) suggesting a link between language skills and the processing of place-value information we hypothesized that children with CI should experience particular difficulties as the demands on place-value processing increase. This is the case for (i) borrow as compared to non-borrow problems in subtraction (requiring to borrow from the tens place depending on the relation of the units) and (ii) for increasing number ranges in the number line estimation task (requiring to integrate three instead of two digits in the 0–1000 compared to the 0–100 range). As regards subtraction we observed that the borrowing effect was indeed more pronounced in children with CI as compared to NH controls. Importantly, this finding is driven by a specific decrease of performance of children with CI for subtraction problems requiring a borrow procedure. Importantly, the hypothesis that this may be associated with the impaired hearing/language experience of children with CI is corroborated when specifically considering the correlation of hearing age of these children with their performance in borrow subtraction problems. The correlation analysis revealed the to-be-expected reliable negative correlation in children with CI [*r*(45) = -0.28, *p* < 0.05, tested one-sided] indicating that with increasing hearing age borrow subtraction problems were solved faster. Moreover, this correlation was not reliable in children without CI [*r*(49) = 0.14, n.s.]. And Fisher’s *Z*-test indicated that the difference between the two correlations was significant (*Z* = 2.01, *p* < 0.05). This is well in line with our argument that hearing and thus language experience of children with CI is specifically related to their place-value understanding as particularly relevant in borrow subtraction problems.

Furthermore, we also found a similar place-value related effect for the number line estimation task. With the increase of the number range from 0 to 100 to 0 to 1000 the estimation error increased more strongly for children with CI than for NH controls. Again, this supported our hypothesis of a specific impairment of children with CI that might result from poorer place-value understanding that, in turn, might originate from their atypical language development. Helmreich et al. (2011) observed that number line estimations of German-speaking children were less accurate than that of Italian-speaking children and attributed this to the way the place-value structure of Arabic numbers is reflected in the respective languages’ number words. While the order of tens and units in symbolic numbers is reflected correctly in Italian number words (e.g., 27 → ventisette, i.e., twentyseven) it is inverted in German number words (e.g., 27 → siebenundzwanzig, i.e., literally sevenandtwenty). This indicates an influence of language representations on number line estimations. In line with this LeFevre et al. (2010) found number line performance to be predicted by the linguistic pathway of their model of numerical development. In turn, this language dependency of place-value processing (e.g., Pixner et al., 2011a,b) might account for the impaired processing in children with CI when demands on place-value understanding increase in both number line estimation in a wider range and borrow subtraction problems.

Nevertheless, it is important to note that subtractions used in the present study were comprised of two-digit operands. According to the literature, language deficiencies (as seen in children with SLI) are often accompanied by working memory impairments (e.g., Henry et al., 2012). Thus, it would be plausible to speculate that children with aberrant language development such as our children with CI experience specific difficulties upon solving borrow subtraction problems because these pose heavy demands on working memory resources. Following this rationale, children with CI (and atypical language development) should be at a clear disadvantage upon solving these kinds of tasks. However, in the present study children with CI were found to have comparable verbal working memory scores and even significantly better CE scores than NH controls. Therefore, the poor working memory hypothesis cannot account for the current results. Additionally, influences of this variable have been accounted for in the analysis. Furthermore, it should be noted that in Austria, children acquire numbers up to 1000 in third grade. Because participating children attended third to fifth grade, one may assume that our results (children with CI performing poorer on large number ranges than controls) are attributable to third graders who do not yet master numbers beyond 100. However, this was not the case. Additional analyses revealed that estimation accuracy did not differ significantly between children with CI attending third grade and those attending fourth and fifth grade [*t*(43) < 0.89; *n.s.*].

## CONCLUSION

Taken together, our results disclose that children with CI (and an associated atypical language development, cf. Boothroyd et al., 1991; Dawson et al., 1995; Geers et al., 2009; Pisoni et al., 2010; Ingvalson and Wong, 2013; Rinaldi et al., 2013) show general as well as specific numerical/arithmetic impairments. On the one hand, an increased number of children with CI was found to show indication of dyscalculia symptoms on a standardized arithmetical test. Additionally, children with CI were generally slower than NH controls in multiplication and subtraction. Finally, also their number line estimations were less accurate. Synced with no differences for the processing of problem and error types in multiplication this seems to indicate that the main impairment of children with CI is a general slowing. However, on the other hand, children with CI exhibited very circumscribed difficulties associated with the processing of place-value information. Performance declined more strongly than for NH controls when (i) subtraction required a borrow procedure and (ii) number line estimation was to be performed within a wider number range requiring the integration of units, tens and hundreds instead of only units and tens. As demands on place-value understanding is increased in both of these cases the language dependency of place-value processing (LeFevre et al., 2010) might account for the impaired performance in children with CI.

It is important to note that we did not evaluate the influence of an atypical language development in children with CI on their numerical/arithmetic abilities by correlating their performance in a language task with those in numerical/arithmetic tasks. Because recent studies consistently found atypical language development in children with CI (e.g., Boothroyd et al., 1991; Dawson et al., 1995; Geers et al., 2009; Pisoni et al., 2010; Ingvalson and Wong, 2013; Rinaldi et al., 2013) we compared the performance of children with CI and a control group in specific numerical/arithmetic chosen for either their strong (i.e., multiplication fact retrieval) or weaker (i.e., two-digit subtraction and number line estimation) dependence on language-related processing. The specificity of the present results corroborated this research strategy. Yet, future studies employing a combined approach are desirable to cross-validate the present findings.

Nevertheless, our findings are original because we were able to show that a large and carefully selected group of children with CI (attending third to fifth grade) displayed overall comparable performance levels and profiles on arithmetic tasks thought to rely heavily on language demands (i.e., multiplication facts). Thus, it seems that despite initially atypical language development, which might account for the general slowing, children with CI are able to acquire arithmetic skills in a qualitatively similar fashion as their NH peers. Nonetheless, with increasing task complexity that is reflected by the necessity to quickly access the positional base-10 place-value system of the Arabic notation, children with CI perform poorer than their NH peers. The present findings have important implications for educational practice and continuing education of children with CI. In particular, our findings suggest that (i) children with CI may perform equally well than their hearing peers provided they are given some extra time to solve arithmetic problems, and (ii) children with CI may require more focused teaching of the Arabic base-10 place-value system in order to make up for their initial (and probably language-related) difficulties to acquire numerical skills.

## Conflict of Interest Statement

The authors declare that the research was conducted in the absence of any commercial or financial relationships that could be construed as a potential conflict of interest.
